# Variations in cag pathogenicity island genes of *Helicobacter pylori* from Latin American groups may influence neoplastic progression to gastric cancer

**DOI:** 10.1038/s41598-020-63463-0

**Published:** 2020-04-16

**Authors:** Cosmeri Rizzato, Javier Torres, Ofure Obazee, Margarita Camorlinga-Ponce, Esperanza Trujillo, Angelika Stein, Alfonso Mendez-Tenorio, Maria Mercedes Bravo, Federico Canzian, Ikuko Kato

**Affiliations:** 1 Department of Translation Research and of New Technologies in Medicine and Surgery, University of Pisa, Pisa, Italy; 2 Unidad de Investigación en Enfermedades Infecciosas, UMAE Pediatría, Instituto Mexicano del Seguro Social, Mexico City, Mexico; 3 Genomic Epidemiology Group, German Cancer Research Center (DKFZ), Heidelberg, Germany; 4 Grupo de Investigación en Biología del Cáncer. Instituto Nacional de Cancerología, Bogotá, Colombia; 5 Laboratorio de Biotecnología y Bioinformática Genómica, ENCB, Instituto Politécnico Nacional, México City, México; 6 Department of Oncology and Pathology, Wayne State University School of Medicine, Detroit, MI USA

**Keywords:** Bacterial genomics, Gastric cancer

## Abstract

*Helicobacter pylori* (HP) colonizes the human stomach and induces acute gastritis, peptic ulcer disease, atrophic gastritis, and gastric adenocarcinoma. Increased virulence in HP isolates derives from harboring the *cag* (cytotoxin-associated genes) pathogenicity island (*cag*PAI). We analyzed the microvariants in *cag*PAI genes with the hypothesis that they may play an important role in determining HP virulence. We tested DNAs from *cagA* positive patients HP isolates; a total of 74 patients with chronic gastritis (CG, N = 37), intestinal metaplasia (IM, N = 21) or gastric cancer (GC, N = 16) from Mexico and Colombia. We selected 520 non-synonymous variants with at least 7.5% frequency in the original sequence outputs or with a minimum of 5 isolates with minor allele. After adjustment for multiple comparisons, no variants were statistically significantly associated with IM or GC. However, 19 non-synonymous showed conventional P-values < 0.05 comparing the frequency of the alleles between the isolates from subjects with gastritis and isolates from subjects with IM or GC; 12 of these showed a significant correlation with the severity of the disease. The present study revealed that several *cag*PAI genes from Latin American Western HP strains contains a number of non-synonymous variants in relatively high frequencies which could influence on the clinical outcome. However, none of the associations remained statistically significant after adjustment for multiple comparison.

## Introduction

Gastric cancer has the third highest mortality rate and the fifth highest incidence worldwide^1^. The two regions of the world with highest mortality rate for gastric cancer are Asia and Latin America accounting for almost two thirds of all gastric cancer deaths^2^. Within the US, ethnic minorities, e.g., Asians, Blacks, Hispanics and Native Americans, experience an incidence almost twice as high as non-Hispanic Whites^3^. Some Asian countries, such as Japan, have nation-wide screening programs for early detection of gastric cancer, whereas most other high-risk countries, such as Latin American countries, do not have such programs, nor does the US for ethnic minorities^4–6^. Although *Helicobacter pylori* (HP) is an established cause of gastric cancer^7^, eradication of HP in general asymptomatic populations has not been advised, because of the large number of persons already infected in high risk populations (>90%), high reinfection rates in endemic areas^8^, antibiotic resistance, high cost of the treatment^9^ and the increased risk of esophageal cancer associated with HP-negative/eradicated individuals^10–12^. Thus, new strategies for gastric cancer prevention are warranted and may help reduce health disparities, mainly in the most affected and underdeveloped regions of the world^13^. All considered identification of new HP variants potentially useful to predict gastric cancer risk will be invaluable not only for vaccine development, but also to target antibiotic treatment to high-risk individuals.

HP has a remarkably high level of genetic diversity due to recombination rates higher than in any other known bacterial species^7,14,15^. A number of HP virulence factors have been identified, but it is now clearly established that *cagA* and the cytotoxin-associated gene pathogenicity island (*cag*PAI) play a central role in the pathogenesis of HP-associated diseases^16,17^. The *cag*PAI consists of a 40 kb chromosomal DNA and is present in approximately 95% of East Asian isolates, compared to 60% of low-risk Western isolates^18–21^. These genes encode cytotoxins and components of the type IV secretion system (T4SS) that acts as a molecular syringe injecting bacterial macromolecules into host cell cytosol^19^. This ultimately leads to sustained IL-8 production, inflammation, proliferation and morphological changes of gastric epithelial cells which underlie HP-induced gastric premalignant and malignant pathologies^19^. However, the presence of *cagA* (a marker of *cag*PAI) does not predict clinical outcomes in high-risk populations since the majority of HP are *cagA* positive strains, and among the infected subject less than 3% develop gastric cancer.

*Cag*PAI contains 31 open reading frames, named *cag1* to *cag26* or *cagA* to *cag*Z and *cag*α to *cag*ζ, or by locus name of the HP 26695 or HP J99 strains genomes^22^. A number of the *cag*PAI genes are homologous to type IV secretion system genes (T4SS) of *Agrobacterium*, *VirB1*-*11, VirD4*, and *VirE1*. In *Agrobacterium tumefaciens* model, the T4SS is composed of 13 proteins, which span both bacterial and host cell membranes^23–26^. Because of the relevance of *cag*PAI in the biological activities of HP that lead to tissue damage, microvariabilities in *cag*PAI genes other than *cagA* are likely to play an important role in determining HP virulence. In this work we aim to study the microvariability of all the cagPAI genes with the exception of *cagY*, for which we limited our analysis to 339 nucleotides at the 3' end of the genes encoding for the last carboxyl terminal 113 amino acids, CagYc)^27^. The present study is meant to extend our previous work based on 454 sequencing^28^ by including new cases with premalignant lesions, and by studying additional cagPAI genes using a newer sequencing platform to increase the power of SNP detection. Data from the present work have been included in a phylogenetic analysis of Latin American HP strains^29^. We performed a comprehensive screening of single nucleotide polymorphisms (SNP) in relation to gastric histopathology in order to identify variants with potential predictive value for clinical outcome, which warrant further validation in larger samples as well as separate functional analyses.

## Results

### Performance of genome-wide sequencing

We prepared sequence libraries and performed whole-genome sequencing on 92 HP strains, including reference strain 26695. Details of the sequencing outcome are included in supplementary table 1. We obtained a total number of reads per samples between 53,427 and 1,366,065. Genomes were assembled, and we found that the percentage of reads that aligned to the reference sequence ranged between 0% and 60.51%. We excluded from further analysis 3 genomes for which no reads could be aligned, 9 where *cag*PAI was absent, 2 strains isolated from the same patient and reference strain 26695 that was analyzed only as quality control (data not shown).

The coverage of the whole genome for the 74 samples selected for further analysis was on average 39.52×(range 6.31×– 114.45×), and coverage for the cagPAI was 85.31x on average (range 11.08×– 466.87×). The number of reads that aligned to the cagPAI per sample ranged between 3201 and 43462. *cagA* gene was missing in 11 of 92 sequenced strains and *cag*γ in one strain.

### Variability by gene

We summarized the genetic variability detected in the twenty-six cagPAI genes in Table 1. For each gene we computed the degree of variability as the number of sites showing a different genotype compared with the reference strain out of the total number of nucleotides in the gene, both as synonymous and non-synonymous variations (causing therefore differences at amino acid level). We assessed a range of variability from 9.54% in *cagF* to 31.22% in *cagA* at DNA level calculated as ratio of polymorphic position to gene length, while the amino acid variability ranged from 1.8% in the *cagE* gene, which is the minimum variation that we found (with the exception of the analyzed region of *cagY* in which we did not find non-synonymous variations), to 17.82% in *cagC* calculated as ratio of non-synonymous polymorphisms to number of amino acid in the translated protein. The number of polymorphisms identified for each gene are summarized in Table 1.

**Table 1:** Overview of genetic variability in genes in HP cagPAI.

Gene	Alternative gene name	Gene length	Synonymous variants N	Non-synonymous variants N	Polymorphic positions in DNA^a^	Non-synonymous selected for analysis^b^
HP0520	*cagζ*	348	26	30	55	12
HP0522	*cagδ*	1446	156	105	277	52
HP0523	*cagγ*	510	93	48	133	23
HP0524	*cagβ*	2247	286	54	333	16
HP0525	*cagα*	993	97	18	117	6
HP0526	*cagZ*	600	48	26	67	9
HP0527	*cagYc*	339	34	0	32	0
HP0528	*cagX*	1570	119	51	186	17
HP0529	*cagW*	1608	126	45	170	16
HP0530	*cagV*	759	72	21	87	9
HP0531	*cagU*	657	45	17	71	0
HP0532	*cagT*	843	94	19	110	8
HP0534	*cagS*	591	44	44	87	16
HP0535	*cagQ*	381	15	24	43	0
HP0536	*cagP*	354	28	20	52	4
HP0537	*cagM*	1131	111	28	136	12
HP0538	*cagN*	921	76	87	161	33
HP0539	*cagL*	714	63	40	95	13
HP0540	*cagI*	1146	104	62	168	22
HP0541	*cagH*	1113	109	44	151	12
HP0542	*cagG*	429	38	25	67	6
HP0543	*cagF*	807	53	22	77	10
HP0544	*cagE*	2953	299	53	348	23
HP0545	*cagC*	348	48	62	98	25
HP0546a		228	23	14	35	4
HP0547	*cagA*	3552	311	620	1109	172

^a^Number of polymorphic positions differ from number of variants because we found that several indel variants span more than one nucleotide.

^b^non-synonymous variants with at least 7.5% frequencies when compared to the reference sequence.

### Comparison of frequencies of polymorphisms showing a differential distribution between gastritis and IM or GC cases

When comparing the frequency of the 520 selected alleles between the isolates from subjects with gastritis and isolates from subjects with IM or GC we found statistically significant differences in allele distribution for three polymorphisms in *cagA* gene (Q/K427R, N467G and V1041T), three in *cagC* gene (V22I, V37I, I45V), one in the *cagE* gene (K981E), one in *cagL* gene (S10F), one in *cagX* gene (G11N), one in *cagS* (G146D), one in *cag*ζ (S35A), three in *cagδ* (V353I, P406L, N407E) and one in *cagβ* (N125A) (Table 2). Furthermore 4 polymorphisms in *cagA* (V52I, G65R, S194F and Q/R427K) showed a significant trend with grade of the disease.

**Table 2:** Polymorphisms in cagPAI genes showing a differential distribution between gastritis and IM + GC cases.

Gene	Nucleotide change	Amino acid change	IM + GC^a^	Gastritis cases^a^	OR (95%CI)	**FisherP-value**
*cagA*	G154A	V52I	0.17	0	14.45 (0.76–273.50)	0.02
*cagA*	G193A	G65R	0.2	0.03	8.00 (0.90–70.92)	0.047
*cagA*	C581T	S194F	0.17	0	14.45 (0.76–273.50)	0.02
*cagA*	A1280G	Q/K427R	0.97	0.7	**12.61 (1.50–105.81)**	0.007
*cagA*	C1279A	Q/R427K	0	0.21	0.06 (0.00–1.06)	0.011
*cagA*	AA1399–1400GG	N467G	0.73	0.42	**3.73 (1.29–10.81)**	0.021
*cagA*	GTT/CCC3121–3123ACC	V/P1041T	0.57	0.3	**3.01 (1.07–8.47)**	0.044
*cagC*	G64A	V22I	0.38	0.14	**3.90 (1.23–12.34)**	0.032
*cagC*	G109A	V37I	0.35	0.11	**4.47 (1.30–15.41)**	0.025
*cagC*	A133G	I45V	0.3	0.08	**4.79 (1.21–18.96)**	0.035
*cagE*	A2941G	K981E	0.08	0.32	**0.18 (0.05–0.72)**	0.019
*cagL*	C29T	S10F	0.51	0.78	**0.29 (0.11–0.80)**	0.027
*cagX*	GG31–32AA	G11N	0.24	0.54	**0.27 (0.10–0.74)**	0.017
*HP0520_cag*ζ	T103G	S35A	0.32	0.11	**3.84 (1.1–13.36)**	0.046
*HP0522_cag*δ	G1057A	V353I	0.78	0.56	**2.9 (1.04–8.06)**	0.048
*HP0522_cag*δ	C1217T	P406L	0.38	0.14	**3.77 (1.19–11.98)**	0.032
*HP0522_cag*δ	AAT1219–1222GAG	N407E	0.38	0.11	**4.87 (1.42–16.72)**	0.013
*HP0524_cag*β	AAT373–375GCA	N125A	0.69	0.92	**0.21 (0.05–0.82)**	0.035
*HP0534_cagS*	GC437–438AT	G146D	0.84	0.60	**3.44 (1.14–10.4)**	0.035

^a^frequency of variant alleles.

**Figure 1:**
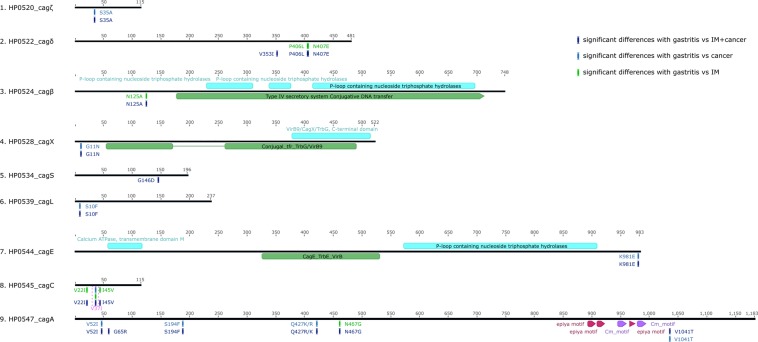
Map of nine *cag*PAI proteins with known functional domains (in green) and the position of 19 amino acid changes derived by non-synonymous SNPs with a statistically significant distribution (P < 0.05) between gastritis and gastric cancer cases (light green); gastritis and intestinal metaplasia gastric cancer cases pooled together (dark blue) and gastritis and intestinal metaplasia cases (light blue).

When IM and GC were analyzed separately and compared with the non-atrophic gastritis, 7 SNPs showed a marginally (P < 0.05) statistically significant association when comparing gastritis vs. IM and 10 when comparing gastritis vs. GC (Table 3). In the *cagA* gene 3 of these variants were associated with risk of IM (V52I, S194F and Q/R427K) and one with GC (N467G), as shown in Table 3. In the *cagC* gene we detected three SNPs with a differential distribution between gastritis and GC (V22I, V37I, I45V, see Table 3). In *cagL* gene polymorphism (S10F) showed a differential allelic distribution between isolates derived from IM cases and gastritis cases (OR = 0.14; 95% CI 0.04–0.46, P = 0.002) (Fig. 1, Table 3). For the *cagX* gene polymorphism G11N showed a differential distribution between cases of IM and gastritis (OR = 0.20; 95% CI 0.06–0.71, P = 0.011) (Fig. 1, Table 3). For *cag*ζ one polymorphism (S35A) showed a differential allelic distribution between isolates derived from IM cases and gastritis cases (OR = 8.80; 95% CI 2.29–33.84, P = 0.001) (Fig. 1, Table 3). In *cagδ* two adjacent polymorphisms showed a differential distribution between cases of cancer cases and gastritis in particular the association for P406L showed an OR = 7.97, 95% CI 2.03–31.27 and for N407E OR = 10.29 95% CI 2.45–43.15. Additionally, the allelic distributions of these two polymorphisms were significantly different between Mexican and Colombian samples (data not shown), namely the variant allele frequencies were extremely low in Colombia, therefore the associations were driven by the Mexican cancer cases.

**Table 3:** Polymorphisms in cagPAI genes showing a differential distribution between gastritis and IM or GC cases.

Gene	Nucleotide change	Amino acid change	Frequency in controls	Frequency in IM	OR (CI)	Frequency in cancer	OR (CI)	P-value for trend
*cagA*	G154A	V52I	0	0.17	**15.13 (0.84–311.2)**^a^	0.17	16.0 (0.71–359.3)	**0.029**
*cagA*	G193A	G65R	0.03	0.17	6.40 (0.61–66.76)	0.25	10.67 (0.99–115.36)	**0.026**
*cagA*	C581T	S194F	0	0.22	**20.79 (1.05–411.83)**^a^	0.08	8.74 (0.33–229.93)	0.110
*cagA*	A1280G	Q/K427R	0.7	1	**16.53(0.91–300.97)**^a^	0.92	4.78 (0.54–42.21)	**0.024**
*cagA*	C1279A	Q/R427K	0.21	0	**0.10 (0.01–1.78)**^a^	0	0.14 (0.01–2.68)	**0.017**
*cagA*	AA1399–1400GG	N467G	0.42	0.67	2.71 (0.82–9.00)	0.83	**6.79 (1.28–35.97)**^a^	**0.010**
*cagA*	GTT/CCC3121–3123ACC	V/P1041T	0.3	0.67	**4.60 (1.35–15.73)**^a^	0.42	1.64 (0.42–6.44)	0.190
*cagC*	G64A	V22I	0.14	0.19	1.51 (0.35–6.36)	0.63	**10.67 (2.68–42.53)**^a^	**0.001**
*cagC*	G109A	V37I	0.11	0.33	**4.13 (1.04–16.37)**	0.38	**4.95 (1.16–21.09)**	**0.019**
*cagC*	A133G	I45V	0.08	0.19	2.67 (0.54–13.29)	0.44	**8.81 (1.89–41.08)**^a^	**0.003**
*cagE*	A2941G	K981E	0.32	0.05	**0.10 (0.01–0.87)**^a^	0.13	0.30 (0.06–1.52)	**0.039**
*cagL*	C29T	S10F	0.78	0.33	**0.14 (0.04–0.46)**^a^	0.75	0.83 (0.21–3.28)	0.310
*cagX*	GG31–32AA	G11N	0.54	0.19	**0.20 (0.06–0.71)**^a^	0.31	0.39 (0.11–1.33)	**0.040**
*HP0520_cag*ζ	T103G	S35A	0.11	0.52	**8.80 (2.29–33.84)**^a^	0.0625	0.53 (0.05–5.19)	0.626
*HP0522_cag*δ	G1057A	V353I	0.56	0.81	3.40 (0.95–12.13)	0.75	2.40 (0.65–8.88)	0.093
*HP0522_cag*δ	C1217T	P406L	0.14	0.24	1.94 (0.49–7.69)	0.5625	**7.97 (2.03–31.27)**^a^	**0.0023**
*HP0522_cag*δ	AAT1219–1222GAG	N407E	0.11	0.24	2.50 (0.59–10.61)	0.5625	**10.29 (2.45–43.15)**^a^	**0.0008**
*HP0524_cag*β	AAT373–375GCA	N125A	0.92	0.76	0.29 (0.06–1.37)	0.6	**0.14 (0.03–0.66)**^a^	**0.008**
*HP0534_cagS*	GC437–438AT	G146D	0.60	0.81	2.83 (0.79–10.21)	0.875	4.67 (0.92–23.79)	**0.028**

^a^P < 0.05 by Fisher Exact test.

In the *cagβ* gene variant N125A showed an inverse association with cancer with an OR of 0.14 (95% CI 0.03–0.66, P = 0.013) (Fig. 1, Table 3).

Next, a multiple comparison analysis was performed by applying a Bonferroni-corrected threshold, and none of the above described SNPs showed a P-value lower than the threshold adjusted for this type of analysis of P = 9.6**×**10^−5^ (0.05/520). None of the SNPs reached this study-wise P-value. Supplementary table 2 lists all the polymorphisms observed in 24 analyzed genes.

### EPIYA and CM motif analysis

We also analyzed EPIYA (A, B or C) and CM (cm) motifs distribution in 72 *cagA* positive sequenced strains^30–32^, while two strains were cagPAI positive but lacking the *cagA* gene. We found a high degree of variability consisting of 12 different patterns, all of the Western Type (supplementary figure 1). Most of the strains (50) presented the A/B/cm/C/cm pattern, followed by the pattern A/B/cm/C/cm/C/cm (in 9 strains), and the pattern A/B/cm/A/B/cm/C/cm/ (in 3 strains). Other less frequent patterns are described in supplementary figure 1. There was no difference in the distribution of the various patterns when compared between the three different disease groups.

## Discussion

This study was conducted in Latin American HP strains, in order to identify specific *cag*PAI micro-variants associated with high-grade gastric lesions. This effort is of high clinical and translational importance as the presence of *cagA* gene does not predict outcomes of HP infection, particularly in high-risk populations where the majority of the strains carry *cag*PAI. The present study not only confirmed the extremely high variability in the *cag*PAI genes, but also pointed to several variants with potential clinical relevance in a few genes for future studies.

Other study have investigated the whole *cag*PAI^33^ however none have performed an extensive analysis of polymorphic variant of each gene in the region.

Overall sequence variability derived from this study for the selected *cag*PAI genes was consistent with that reported previously using different sequencing techniques^28,34^, and extend the study to a higher number of genes. These results support the signature of diversifying selection through bacterial evolution in the proteins that are surface-exposed and involved in interactions with host molecules^34^. However, the frequencies of amino acid variants in *cagA*, *cagC* and *cag*γ found in this study were substantially higher than those previously reported^28,34^. This may be partially due to a greater number of strains under investigation in our work compared to previous publications. While we cannot completely rule out artifacts from this high throughput platform, such artifacts should affect both synonymous and non-synonymous variants.

In a previous study conducted with amplicon sequencing by 454 for 84 Mexican and 11 Venezuelan samples we reported 10 non-synonymous SNPs with differential allelic distribution between gastritis and gastric cancer at conventional P-values between 0.01–0.05^28^. In the present project that included equal numbers of Mexican and Colombian strains we did not see any disease association with these 10 variants. Although variant frequencies were not markedly different between Mexico and Colombia strains, particularly for variants showing a significant association with IM + GC, we have previously reported important phylogenetic differences between strains of these two countries^29^. These phylogenetic differences may partly explain discordant results between the previous and current studies.

Previous publications reported an association of GC with the presence of variants in position 58 and 59 of cagL protein; in two studies^35,36^ the concurrent presence of tyrosine (Y) in amino acid position 58 and glutamic acid (E) in position 59 (Y58E59) compared with the combination aspartic acid (D58) and Lysine (K59), induced more efficiently a shift of gastric integrin a5b1 in the corpus, which has been related with gastric carcinogenesis. In our previous publication we did not observe the Y amino acid in position 58 in any sample, although we did find that carriers of D at this position are at lower risk of GC in comparison with the asparagine (N) carriers^28^. In the current work we confirmed the absence of polymorphism in Y position 58 and the presence of N58D polymorphism. We also observed the E59K polymorphism but we did not find association with either IM or GC in our populations. It should be noted that there was a major difference between our current and previous studies^28^ in the composition of geographical origins of the samples. Our former study included Colombian while the current study included Venezuelan strains; this is relevant considering our recent report where we document adaption of HP genome to different Latin American populations^37^. Furthermore, Gorrel and co-worker^38^ performed an expanded analysis of this region analyzing the sequence from amino acid 58 to 62 and found significant differences according to the geographical origin; in this sense, we confirmed the predominance of the DKMGE aminoacid sequence.

Some of the variants may warrant further studies as the SIFT program^39^ predicts them to be damaging non-tolerant. In particular the *cagL* S10F variant (located at codon 10) changes from serine (polar) to phenylalanine (non-polar). Interestingly, among the 43 Asian strains recently sequenced, no single S10F variant was found, suggesting that this is a Western-strain specific variant^35^. Thus, overall, the differences observed in these studies are likely to be driven by geographical origins of HP.

The other variant that is considered to be detrimental non-tolerant is located at residue 11 of *cagX*, exchanging glycine (non-polar) to asparagine (polar). cagX, Vir9 homologous protein, has been found recently to be necessary for the formation of HP pilus^40^, mediating the stabilization of cagT which is a T4SS structural protein^41,42^. Thus, cagX mutants prevent cagA biological activities^40^. In this context, our finding of a protective effect of the N allele is compatible with a lesser virulent behavior.

One *cagC* variant, located at codon 22, replacing valine with isoleucine, was associated with risk of gastric cancer and predicted to be intolerant by SIFT. Valine to isoleucine substitutions have been reported to result in changes in protein structure, kinetics and stability in both bacteria^43–45^ and humans^45–47^. However, the possible function of this polymorphism remains unclear.

The four polymorphisms in genes *cag*ζ, *cag*δ and *cag*β showing a different distribution in IM or cancer cases were predicted to be tolerated by SIFT.

There were several variants at the N-terminal of *cagA* that showed rather strong (OR > 4.60) associations with high grade lesions (P = <0.025) (Table 2) as well as a significant trend by type of lesions (P < 0.05) (Table 3). Some involved significant changes in amino acid characteristics (e.g., Q427R, N467G), although none were predicted to be detrimental by SIFT. *cagA* N-terminal region (residue 1–884) has recently received intensive research interest owing to its ability to interact with exogenous molecules, including host tumor suppressors, adhesion molecules, inflammatory mediators as well as chemopreventive agents such as curcumin^48,49^. Further characterization of *cagA* N-terminal region may shed light on potential function of the variants found in this study.

Strengths of this work include the relatively large number of HP samples completely sequenced. This work adds significantly to the number of HP complete genomes publicly available, and it substantially increases the available number of HP genomes from Latin America, spanning also different stages in the natural history of HP infection and progression to gastric cancer. These new data have already been used for an in-depth phylogenetic analysis of Latin American HP genomes^29^. Additionally, the sequences used in the final analysis are of high quality, with an average coverage of about 40×, which is more than enough for a thorough assessment of sequence variation.

On the other hand, we acknowledge some limitations in this study, which was designed as a first stage to screen candidate *cag*PAI variants to be validated in a larger study, and sample size felt short to obtain reliable risk estimate for high-grade gastric lesions, particularly when IM and cancer were considered separately. In the same vein, the sample was too small to assess combinations of several potentially interesting variants. Also, our study did not include Asian strains that present marked differences in the CagA EPIYA region, and thus our results do not apply to Asian strains. Furthermore, our sequencing platform HiSeq was not suitable to analyze long repeat regions such as those in *cagY*, which are rather common in Hp bacterial genome. That is a major reason why we limited *cagY* analysis to its conserved region (Yc). Finally, our data were exclusively based on cultivable HP from gastric biopsies. Little is known as to whether bacteria that are easy to grow *in vitro* are genetically different from those that are difficult to grow *in vitro* but able to survive in the human stomach for extended periods of time.

Despite the several limitations discussed above, the present study revealed that several *cag*PAI genes from Latin American Western HP strains contain a number of non-synonymous variants in relatively high frequencies. Some of these variants warrant further investigation to better understand their clinical significance in larger association studies, as well as experimental studies to elucidate their biological functions, and bioinformatic analysis to gain structural insights of the sequence variants.

## Methods

### Study population

Strains analyzed in this study were isolated from patients recruited in the context of a multi-centric study based in Latin America^50,51^. Sequences used in the present work are largely overlapping with those reported in a phylogenetic analysis on Latin American HP strains^29^. Patients attended the gastroenterology or oncology services and were subjected to endoscopy for diagnostic purposes. We isolated HP in 92 of these patients and sequenced them, however 18 were dropped due to poor quality or because they did not carry the *cag*PAI. Samples included in the following analysis were therefore 74 HP clinical isolates from 74 individuals recruited in Colombia (N = 37) and Mexico (N = 37, nine of which have been already sequenced with the 454 technology for 5 genes^28^). Thirty-seven of these subjects had non-atrophic or atrophic gastritis, 21 intestinal metaplasia (IM) and 16 distal gastric cancer (GC). Table 4 shows pertinent characteristics of the population. For Mexican samples, all the patients signed an informed consent and the study was approved by ethical committees of the Instituto Mexicano del Seguro Social (IMSS) and General Hospital of the Secretaria de Salud (SS), Mexico City, Mexico^28^. For Colombia, the clinical studies where patients were originally recruited were approved by the Ethical and Research Committee of the Instituto Nacional de Cancerología, and all the patients signed an informed consent. This study was approved by the Ethical and Research Committee of the Instituto Nacional de Cancerología^52^. All research was performed in accordance with relevant guidelines and regulations.

**Table 4:** Characteristics of the study population.

	Number of samples	Colombian	Mexican	Total
Diagnosis	**Gastritis cases**	21	16	37
	**Metaplasia cases**	12	9	21
	**Cancer cases**	4	12	16
Gender	**Female**	13	24	37
	**Male**	22	13	35
	**Unknown**	2		2
Median age (25%–75%)	**Gastritis cases**	43 (37–59)	45.5 (37.5–55.5)	
	**Metaplasia cases**	51.5 (46–59)	58 (52–68)	
	**Cancer cases**	70 (62.5–70)	53 (46–59)	

### Sample preparation

In order to isolate HP, Mexican stomach biopsies were homogenated and inoculated on 5% sheep Blood agar base (Becton Dickinson, New Jersey, USA) supplemented with vancomycin, trimethoprim, polymyxin B (Campylobacter-selective antibiotics, Oxoid, LTD. England). Colombian biopsies were homogenated and cultured on blood agar plates, supplemented with Campylobacter-selective supplement (Oxoid), 1% Vitox (Oxoid), 7% horse serum (Invitrogen). Plates, in both centers, were incubated at 37 °C under a 10% CO_2_ atmosphere, and genomic DNA was extracted from HP colonies using the DNeasy Mini Kit (Qiagen, Hilden Germany).

### Whole-genome HP sequencing

Nextera XT sample preparation kit (Illumina) was used for preparation of the sequencing libraries according the manufacturer’s instructions. 1 ng of dsDNA libraries was quantified with Picogreen and used as input for Illumina sequencing. High Sensitivity DNA Kit (Agilent Technologies) was used to verify the fragment length distribution of the libraries using the agilent Bioanalyzer. Sequencing was performed on an Illumina HiSeq. 2500 sequencer with v4 PE125 chemistry at the Genomics and Proteomics Core Facility of DKFZ.

### Bioinformatic and statistical methods

Raw sequences with Phred score >30 were analyzed with FastQC (https://www.bioinformatics.babraham.ac.uk/projects/fastqc/) to further assess quality. The resulting sequencing output, in fastq format, was assembled with the “map by reference” function of the Geneious software platform (http://www.geneious.com/), considering the sequence of the 26695 strain (NC_000915.1) as reference. A consensus sequence was determined for *cagA* (HP0547), *cagC* (HP0546), *cagE* (HP0544), *cagF* (HP0543), *cagI* (HP0540), *cagL* (HP0539), c-terminal sequence of *cagY* (HP0527) (339 nucleotides at the 3’ end of the genes encoding for the last carboxyl terminal 113 amino acids of CagY, *cagYc*)^28^, *cagX* (HP0528), *cag***γ** (HP0523), and single nucleotide polymorphisms and small insertions and deletions were identified for each gene. For the analysis of *cagA* gene an additional strategy was used to better analyze the EPIYA and CM motifs (C-MET motif mediate CagA multimerization and membrane targeting)^30,31^. Illumina reads of the *cagA* gene were extracted and realigned by the “de novo assemble” option of the same software. To exclude potential artifacts in sequencing and to enrich variants with clinical relevance, we selected a total of 520 non-synonymous variants with at least 7.5% frequency in the HP isolates we included in the analysis or with a minimum of 5 isolates with the variant allele. A Bonferroni-corrected threshold (P = 0.05/520 = 9.6**×**10^−5^) was used to adjust for multiple comparisons. The variant alleles were determined using strain 26695 as reference. When more than one variant resulted in substitution of the same amino acid, we also analyzed the frequencies of the combined amino acid variant. We used non-atrophic gastritis as the control group to compare frequencies of variants in IM and GC groups or a combined group with the two pathologies, and genotypes at a given locus were dichotomized, a selected variant vs. all other genotypes. P-values for differences in allelic frequencies between the control and IM/GC combined or separately were determined by the Fisher’s exact test (2-sided). We also computed odds ratios (OR) 95% confidence interval (CI) for these gastric pathologies using logit estimators to obtain the CI even for the variants with zero frequencies in any category. For the variants that showed an unadjusted Fisher P-value of <0.05, we further tested linear trends of their frequencies across the three histological groups, control, IM and GC, using Mantel-Haenszel chi-square test. These analyses were performed by SAS version 9. The effect of DNA polymorphisms on the predicted proteins was evaluated with a bioinformatics tool: SIFT (**S**orting **I**ntolerant **F**rom **T**olerant) http://sift.jcvi.org^53^.

## Supplementary information

Supplementary figure 1.

supplementary table 1.

supplementary table 2.

## Data Availability

All sequences from Mexican strains are deposited at DDBJ/ENa/GenBank under the bioprojects PRJNA338771 and PRJNA203445. Genome sequences from Colombia are deposited under the bioproject PRJNA352848. GenBank accession numbers for 69 out of 74 analyzed strains are reported in supplementary table 1, submission of the remaining strains to GenBank is ongoing. In the meantime, the data are available upon request to the corresponding author.
