# Supplementary material for: Variations in cag pathogenicity island genes of Helicobacter pylori from Latin American groups may influence neoplastic progression to gastric cancer
Source: Sci Rep. 2020 Apr 16;10:6570. doi: 10.1038/s41598-020-63463-0 (PMC7162905; doi:10.1038/s41598-020-63463-0)

Variations in cag pathogenicity island genes of *Helicobacter pylori* from Latin American groups may influence neoplastic progression to gastric cancer

Cosmeri Rizzato[1*], Javier Torres[2], Ofure Obazee[3], Margarita Camorlinga-Ponce[2], Esperanza Trujillo[4], Angelika Stein[3], Alfonso Mendez-Tenorio[5], Maria Mercedes Bravo[4], Federico Canzian[3], Ikuko Kato[6]

[1] Department of Translation Research and of New Technologies in Medicine and Surgery, University of Pisa, Pisa, Italy
[2] Unidad de Investigación en Enfermedades Infecciosas, UMAE Pediatría, Instituto Mexicano del Seguro Social, Mexico
[3] Genomic Epidemiology Group, German Cancer Research Center (DKFZ), Heidelberg, Germany
[4] Grupo de Investigación en Biología del Cáncer. Instituto Nacional de Cancerología, Bogotá, Colombia
[5] Laboratorio de Biotecnología y Bioinformática Genómica, ENCB, Instituto Politécnico Nacional, México City, México
[6] Department of Oncology and Pathology, Wayne State University School of Medicine, Detroit MI, USA

Corresponding author:
*Dr Cosmeri Rizzato
Department of Translation Research and of New Technologies in Medicine and Surgery
University of Pisa
Pisa, Italy
cosmeri.rizzato@unipi.it

Supplementary figure 1. Schematic alignment of the different patterns of EPIYA A, B or C motifs and CM motifs with number of observations in 72 sequenced Mexican and Colombian isolates. This analysis has been performed by extraction of Illumina reads of the *cagA* gene and realignment by the "de novo assemble" option of the same software. Subsequently, the consensus sequence of each isolate has been translated into amino acid sequence and aligned again.

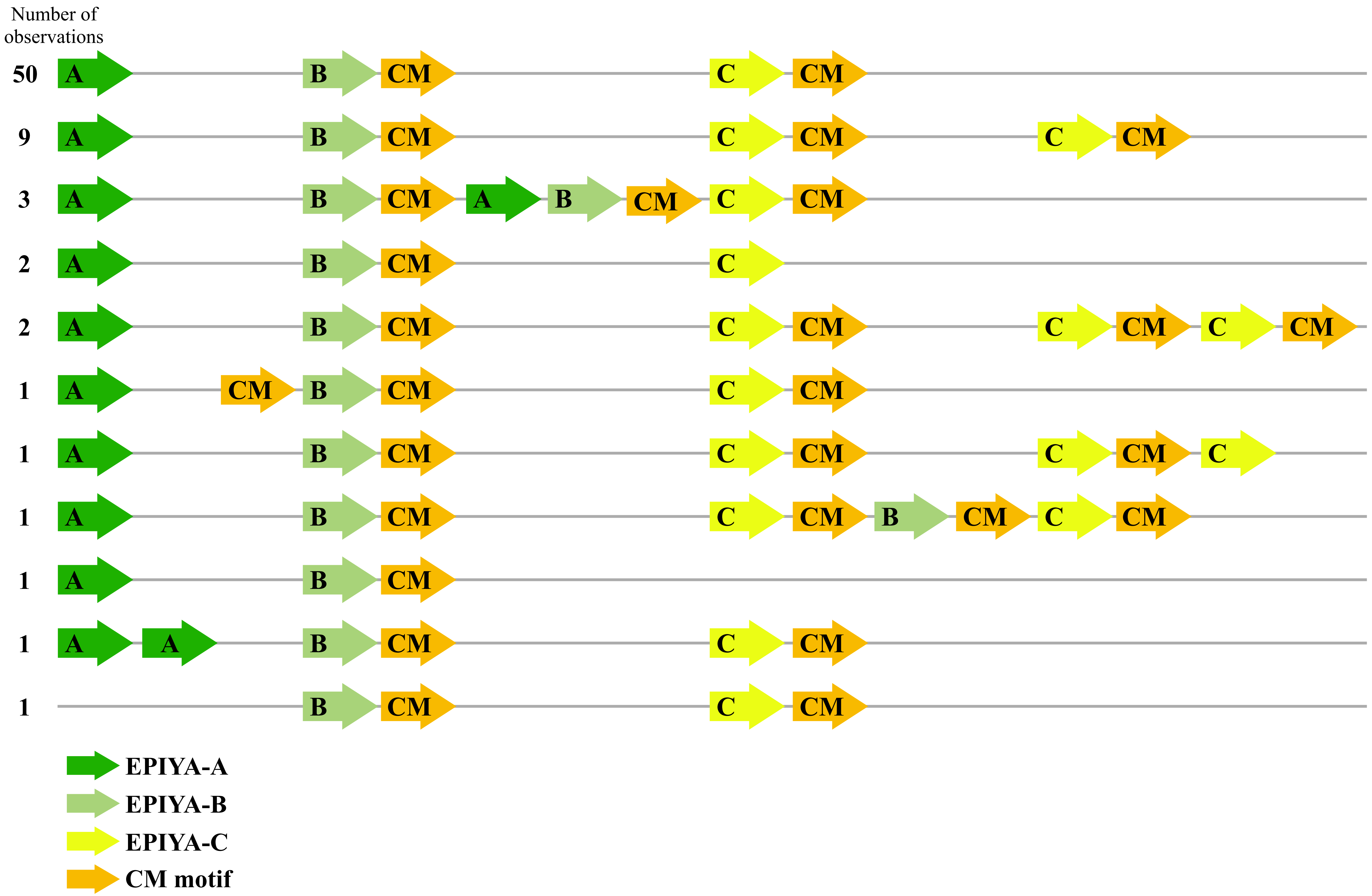

Supplement: Supplementary file 1 — Supplementary figure 1. [file 41598_2020_63463_MOESM1_ESM.pdf]
